# A Qualitative Study on the Recovery Process and Its Associated Factors in Morita Therapy for Inpatients with Mood Disorders

**DOI:** 10.3390/jcm12186016

**Published:** 2023-09-17

**Authors:** Kazuo Tanii, Mikiko Kubota, Kei Nakamura, Akihiko Nunomura, Masahiro Shigeta

**Affiliations:** 1 Department of Psychiatry and Center for Morita Therapy, The Jikei University Daisan Hospital, Komae-shi, Tokyo 201-8601, Japan; 2 Department of Psychiatry, The Jikei University School of Medicine, Minato-ku, Tokyo 105-8461, Japan; 3 Graduate School of Social Well-Being Studies, Hosei University, Machida-shi, Tokyo 194-0298, Japan

**Keywords:** Morita therapy, mood disorders, inpatient, recovery process, subjective experiences

## Abstract

Morita therapy (MT) has been re-evaluated and has attracted much attention internationally to date. However, it is not known what kinds of experiences inpatients with mood disorders undergo during the process of recovery through MT. The purpose of this study was therefore to empirically clarify what subjective experiences influence the recovery from depression when it is treated with MT. Patients with mood disorders who were admitted to the Jikei University Center for Morita Therapy were included. Successive assessments of depression were performed using rating scales. Semi-structured interviews were conducted at the time of discharge regarding factors contributing to improvement, and were analyzed using qualitative data analysis methods to identify factors contributing to the recovery from depression among inpatients treated with MT. There were 24 subjects, 19 of whom completed treatment. The completers had significantly lower severity of depression severity upon discharge. Remarkably, qualitative analysis revealed that nine categories of experiences contributed to recovery from depression. In particular, experiences of “isolation bed-resting of MT”, “getting stuck in doing things one’s way”, “identifying maladaptive behavior patterns”, “modifying maladaptive behavior patterns”, “restoring self-evaluation”, and “change in negative emotions” were considered as the key experiences for recovery.

## 1. Introduction

Morita Therapy (MT) is an original psychotherapy developed by Japanese psychiatrist Shoma Morita in 1919 [1], which has been re-evaluated and has attracted much attention internationally to date [2,3,4,5,6,7,8,9,10,11].

Although MT is a psychotherapy for neuroses such as anxiety disorders, obsessive–compulsive disorder, and hypochondriacal disorder, it has also been used to treat depressed patients since the time of Morita [12]. He regarded certain cases of periodic depression as an impure subtype of neuroticism, and he located its psychopathology in the mechanism of fixation and development (a vicious cycle) due to psychic interaction, based on anticipatory fear and self-suggestion. Subsequently, Nakamura [13] advocated yojo (several ways of taking better care of oneself) for depressed patients, based on the key concept of MT, “arugamama (accepting it as it is)”. This method involves, first, accepting the reality of suffering from depression; adjusting one’s daily life according to the stage of recovery so as not to cause a vicious cycle; and gradually shifting from rest to activity in the convalescence stage to encourage the healthy functioning of the body and mind by naturally exercising the “desire for life”. Yojo, as above, with a focus on activities using the environment of inpatient MT, enables patients to acquire the attitude required for curative purposes through practice, which has a more direct effect than outpatient MT [13].

Outside of Japan, there are reports of pilot randomized controlled studies of outpatient MT for depression that show higher remission and treatment response rates in the combined MT group than in the usual treatment group [9]. In addition, a meta-analysis report showed that outpatient MT in combination with pharmacotherapy reduced depression severity and increased remission rates [5].

Thus, while we have seen some empirical studies and reports suggesting the efficacy of outpatient MT for depression, inpatient MT for depression has been discussed only in a few articles based on inferences drawn either from a small number of patients or based on the clinical experiences of the authors. Therefore, it has not been clarified what kind of changes actually occur with inpatient MT, what kinds of experiences lead to these changes, and how they lead to recovery.

The purpose of this study was therefore to empirically clarify what kind of subjective experiences influence the recovery from depression in the recovery process of patients with mood disorders treated using inpatient MT.

## 2. Methods

### 2.1. Subjects

Patients admitted to the Jikei University Center for Morita Therapy between March 2010 and November 2017, including males and females aged between 20 and 65 years, were eligible for participation in the study. We conducted a single-center study in our university hospital because it was crucial for our qualitative study to maintain quality assurance concerning admission, treatment, and follow-up. The age of the subjects was set at 20 years or older, when growth and development were presumed to be complete, and no older than 65 years, because the subjects were considered to be more susceptible to organic changes after the age of 66 years. Patients were included if they fulfilled any of the following criteria of ICD-10 [14]: mild or moderate depressive episode; recurrent depressive disorder; mild or moderate current episode; bipolar affective disorder; current episode of mild or moderate depression; or dysthymia. Patients were excluded if they presented other concomitant mental or behavioral disorders, depressive states with symptomatic or organic causes, or if they were readmitted to the hospital.

Subjects were diagnosed using ICD-10 [14] at the time of admission, and any comorbid psychiatric disorders (e.g., anxiety disorders) were also diagnosed using ICD-10 [14] at the time of admission, and subjects were excluded if they had comorbid disorders. Symptomatic or organic causes were ruled out via neurological examination, blood biochemical tests including thyroid function, head MRI or CT scans, and electroencephalography. Examples of symptomatic and organic causes of depression that were excluded include thyroid dysfunction and cognitive decline with brain atrophy.

### 2.2. Interview on Admission

The patients were interviewed on admission about their current medical history, medication, and past medical treatment.

### 2.3. Sequential Assessment of Depression

Using the Japanese version of the Hamilton Depression Rating Scale (HAM-D) [15], the authors conducted assessment interviews on admission and every two weeks thereafter to assess severity of depression over time. The patients were asked to self-evaluate using the Japanese version of the Beck Depression Inventory-Second Edition (BDI-II) [16] on admission and every week thereafter for successive assessment.

### 2.4. Interview at Discharge

Semi-structured interviews were conducted within one week of the discharge date to confirm medication details and to identify factors contributing to improvement. The questions asked in the semi-structured interview consisted of the following: (1) When do you think your depressive state started to improve? (2) What changes did you notice that caused you to feel the improvement? (3) Were there any events that triggered the improvement? What was it like? (4) (While sharing the BDI-II and HAM-D graphs with the patients) Was there anything that happened at the inflection point (defined as the point where both scores showed significant improvement)? What did you experience around that time? (5) Besides (3) and (4), was there anything else that helped you to recover from depression? Tell us in more detail about the experience.

The patients were asked to answer the above questions freely, and more questions were added as needed. The interviews were conducted by the authors, and were recorded on a voice recorder, and these recordings were converted into text data. In addition, the nursing logbooks and the daily diaries written by the patients over the course of their inpatient treatment were used to confirm the timing of their experiences.

The data obtained via the above methods (subjective experiences leading to improvement) were analyzed using the qualitative data analysis method [17]. First, the content of the text data was read to find the relevant phrases, and the contexts of the descriptions related to the experiences that contributed to recovery were extracted and used as sentence segments. The segments were coded, focusing on their similarities and differences, and categories were created. In addition to examining the contents of the categories, the relationships among the categories were examined to extract the factors contributing to recovery from depression using inpatient MT.

This study was conducted to elucidate the factors that contribute to recovery from depression with inpatient MT, and it was considered useful to use a qualitative research method that can examine the subjects’ subjective experiences in detail, which cannot be adequately captured by existing quantitative research methods using questionnaires. In order to ensure the reliability and validity of this study, the analysis and examination of the data were carried out by three evaluators, namely, the first author; a clinical psychologist with experience in qualitative research (36 years of experience); and a psychiatrist (with 41 years of clinical experience).

### 2.5. Inpatient MT at the Jikei University Center for Morita Therapy

Inpatient MT consists of four stages, and the standard inpatient treatment nowadays takes approximately three months, compared to 40 days with the original Morita method. The following is an overview of the inpatient treatment at the Jikei University Center for Morita Therapy that the subjects underwent.

1. Stage 1: Isolation bed-resting period (usually 1 week)

In this stage, patients spend the entire day in bed except for eating, washing, and using the toilet. Therapists make brief rounds once a day. The aim during this period is to stop trying to eliminate symptoms and for the patients to face their own conditions.

2. Stage 2: Light occupational work period (usually 5 days)

During this period, patients perform light tasks that can be done on their own. In practice, the activities include visits and observations inside and outside of the wards, woodcarving and pottery making. The aim is to encourage spontaneous activity and to develop an attitude of not being carried away by moods and of engaging in what is in front of them. They also start to participate in consultation sessions with therapists (2–3 times a week) and diary guidance during this period. They are asked to write down what they did that day and their living conditions in their diaries, on which the therapists provide comments.

3. Stage 3: Intensive occupational work period (usually lasting about 2 months)

In this stage, patients cooperate with other patients to carry out various basic daily tasks, such as cleaning the ward and taking care of plants and animals. Since it is important that they work spontaneously in occupational therapy, patients decide which tasks they engage in. Later in this period, they take on a responsible role in the overall work of the ward. Thus, the aim is to cultivate an attitude facilitating flexible actions, regardless of symptoms, through the practice of daily living

4. Stage 4: Preparation for daily living period (usually 1 week to 1 month)

This is the stage for patients to think concretely about their life after discharge and to prepare to return home. This may include overnight stays at home and, if necessary, commuting from the ward to work or school, just as in a night hospital.

### 2.6. Ethics

The present study was conducted with the approval of the Ethics Committee of the Jikei University School of Medicine for Biomedical Research (Document number: 21-181:6059). Eligible participants were introduced to the aim, procedures, perspectives and the possible discomforts or benefits associated with the study. They signed a standard informed consent form describing their rights to withdraw at any time during the study without this having any consequences for their treatment in general.

### 2.7. Statistics

The background of the subjects was indicated by the frequency of the category for nominal variables, and the mean ± standard deviation (SD) was calculated for continuous variables. Student’s two-sided *t*-test was used for comparisons between completers and discontinuers for HAM-D and BDI-II scores. The BDI-II scores measured over time were analyzed using repeated analysis of variance (ANOVA), with a significance level of 5% two-sided, and Bonferroni correction was used to adjust for the multiplicity of tests. Corresponding *t*-tests were used to analyze HAM-D scores and the mean antidepressant dose (imipramine equivalent) at admission and at discharge. Changes over time were illustrated using box-and-whisker plots. The datasets analyzed during the current study are available from the corresponding author upon reasonable request.

## 3. Results

### 3.1. Background of the Subjects

From March 2010 to November 2017, 24 patients (11 women and 13 men) were included in the study, with a mean age of 42.21 ± 9.76 (SD) years. The participants’ diagnoses included two cases of a depressive episode, 13 cases of recurrent depressive disorder, and nine cases of bipolar–affective disorder (all bipolar II disorders), and all subjects met the criteria for a mild or moderate depressive episode. Details are shown in Table 1. Nineteen patients completed treatment (completers), three discontinued treatment and two discontinued the periodic questionnaires at their own request. Completers spent 7–10 days in the isolation bed-resting period, 5–7 days in the light work period, 16–147 days in the intensive work period, and 0–38 days in the preparation for daily living period, receiving a total of 28–170 days of inpatient MT. The isolation bed-resting stage lasted 7 days for 18 patients and 10 days for only 1 patient. The light work stage lasted 5 days for 18 patients and 7 days for only 1 patient. The patient who spent 10 days in the isolation bed-resting period was the one who spent 7 days in the light work period, and these two periods were prolonged at the discretion of the psychiatrist in charge due to the patient’s decreased motivation and general malaise. Five patients were discharged from the hospital after finishing the work period without going through the preparation for daily living period, and the inpatient treatment for all five of these patients turned out to be short-term, lasting about a month. For these five cases of short-term inpatient treatment, the length of hospitalization was limited from the beginning due to financial factors and the length of leave from work. The average antidepressant dose (imipramine equivalent) of completers was 179.2 mg/day on admission and 163.4 mg/day on discharge, indicating no significant difference. The HAM-D and BDI-II scores at admission for completers and discontinuers were compared, but no significant differences were found.

### 3.2. Quantitative Assessment of Depressive States

**Figure 1:**
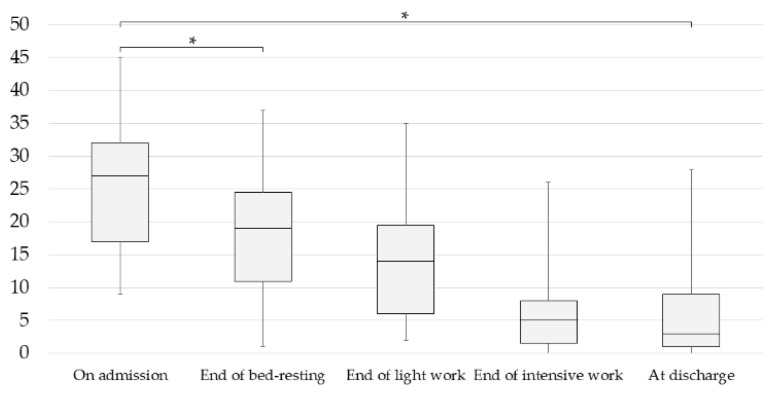
Changes in BDI-II (Beck Depression Inventory Second Edition) scores of completers during inpatient treatment. Data are means with standard deviations (* *p* < 0.05, repeated analysis of variance (ANOVA)). Admission: 25.53 ± 9.18; end of bed-resting: 18.00 ± 9.53; end of light work: 13.89 ± 8.97; end of intensive work: 6.16 ± 6.38; discharge: 5.63 ± 6.63. *p*, Bonferroni-adjusted *p*-value.

**Figure 2:**
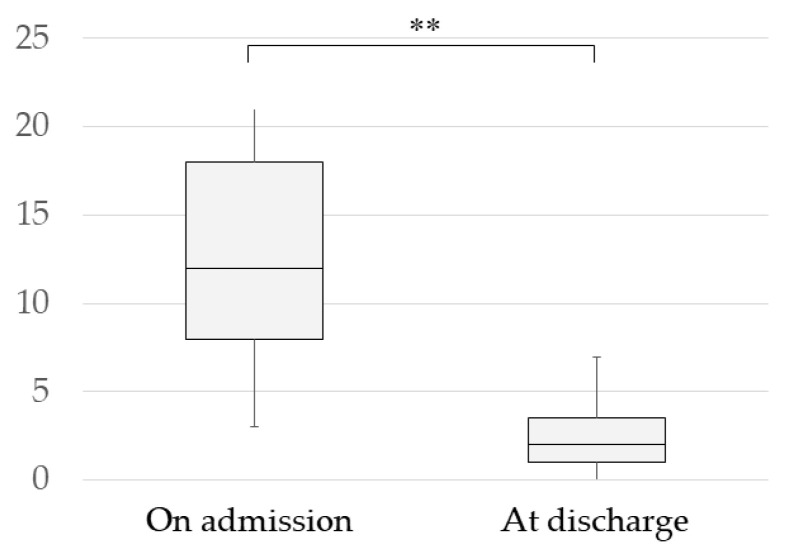
Change in HAM-D (Hamilton Depression Rating Scale) Scores of completers. Data are means with standard deviations (** *p* < 0.01, paired-samples *t*-test). Admission: 13.37 ± 5.86; discharge: 2.63 ± 2.03.

The progression of the BDI-II scores on admission, at the end of the isolation bed-resting period, at the end of the light work period, at the end of the intensive work period, and at discharge from hospital in the completers, is shown in Figure 1. As the HAM-D scores were assessed every 2 weeks and did not coincide with the end of each period, Figure 1 shows the progression of the BDI-II scores only. BDI-II scores on admission were compared with those at the end of the bed-resting stage and at discharge, and both showed a significant decrease (Figure 1). In addition, a comparison of HAM-D scores on admission and at discharge showed a significant decrease in scores at discharge (Figure 2). Moreover, significant reductions were also observed in many subscores of the HAM-D and BDI-II (Table 2 and Table 3). In addition, comparing BDI-II scores on admission and at the end of the bed-resting stage, significant reductions were found in many BDI-II subscores (Table 3). None of the 19 patients changed their medication during this bed-resting period. The score improved in 16 patients and worsened in 3 patients after bed-resting. However, in all three cases in which the score worsened after bed-resting, the BDI-II score at discharge decreased (Case 2: 16 on admission, 25 at the end of bed-rest, 9 at discharge; Case 4: 27 on admission, 29 at the end of bed-rest, 0 at discharge; Case 19: 17 on admission, 25 at the end of bed-rest, 12 at discharge).

### 3.3. Qualitative Research on Factors Contributing to Recovery from Depression

Data (subjective experiences leading to improvement) from the discharge interviews of the completers were analyzed using the qualitative data analysis method [17]. First, the contexts of descriptions related to experiences that contributed to recovery were extracted and coded as sentence segments, paying attention to their similarities and differences, and categories were created (Table 4). In all cases, the experience of isolation bed-resting was described as helpful for recovery. Moreover, as significant improvement in BDI-II scores after bed-resting was observed, the categories of recovery factors were divided for examination into “experience of bed-resting” and “experience after bed-resting”. A categorization of the experiences while bed-resting is shown in Table 5. In addition, experiences as a trigger for improvement, and experiences at the inflection point of improving BDI-II and HAM-D scores, are shown in Table 6.

The specific coding process is shown below using Case 7 and Case 17 (codes are shown in brackets, e.g., {Rest}, etc.

⮚ Case 7
  - <Experience of Bed-Resting>
  I was able to rest in bed {rest}. While bed-resting, it was impressive that I realized I could not find an answer even if I thought it through {thinking it through to find that there was no answer}, and I felt like one of my depressions had gone away {depression gone}. I was thinking about the mistakes I had made in the past and what I should have done then {reflection on the past}. When things did not go well, I often felt that it was always my fault {criticizing oneself}. Around the fifth day, I became bored {bored}, and felt like moving my body as soon as possible {wanting to move my body}. On the last day, anticipatory anxiety became stronger wondering whether I would be able to get along with the others the next day {anxiety about interpersonal relationships ahead} and to do the work {anxiety about the work ahead}. I had a sense of achievement when I finished {sense of accomplishment}. On the first day after bed-resting, the weather was nice and I felt very good {good weather and pleasant}. I was able to concentrate on the light work too {concentrated on light work}.
  - <Experiences after the bed-resting stage>
  When I was very depressed and wanted to take a break {to take a break from work feeling depressed} (omitted), I thought about how I could work and came up with the ideas that I could leave the role of plant leader to others and work on the tasks that I might be able to do no matter how bad my depressive feelings were. As I engaged quietly in the work, I gradually forgot about my depressed mood {mood changes while engaging in tasks in front of me}. To this day, I could not show the incapable part of myself {realizing one’s own tendency}. I had a sense that being perfect was the bottom line {realizing one’s own tendency}. I realized that as I thought I was trying to help everyone become intimate and was taking a role to manage the situation smartly in front of everyone, I became the odd one out without noticing {realizing one’s own tendency}. As we were working as a team, it was a good experience for me that I had to work as everyone else does according to the same schedule so that I could not do “not today” {dividing activity by time}. The experience of being a coleader within a time limit was good. Although I felt, “I should and want to do this and that too”, I realized that there was a limit to time as well as my stamina, and it was a good experience for me to manage within the time limit {modifying one’s own tendency}.
⮚ Case 17
  - <Experience of Bed-Resting>
  I felt rested in bed-resting {rest}. During the day, I thought about the past and regretted it {thinking about the past}, {regret about the past}, and I had a hard time worrying about life after discharge and the future {anxiety about life after discharge}. I had always been anxious about the future {vague anxiety about the future}. In the end, I thought that no matter how much I thought about it, it wouldn’t change my anxiety {thinking it through to find that there was no answer}. Taking a bath on the seventh day of bed-resting felt very good {bathing was pleasant}. Observing the work with pigeons was very interesting {becoming interested in the work}. I could concentrate on wood carving {concentrated on wood carving}.
  - <Experiences after the bed-resting stage>
  About a month and a half into the work period, I became able to do a certain amount of work, and I started to think that I could do so many things, whereas before I thought I was useless because I couldn’t do anything {feeling that I was not useless}, {I felt confident as I could do things}. I was overdoing any kind of work, thinking “I have to do it perfectly” {realizing one’s own tendency}. Since I wanted to do things the way I wanted and feared that if I relied on others, they would think of me as incapable, I always tried to do many tasks on my own and became tired {getting tired doing many tasks alone}. Listening to the advices from the doctor, I gradually started to consult with others and actually relied on others, and I felt relieved and thought that it was alright {modifying one’s own tendency}. I usually overdo things, but here, when it’s time for a break, I can’t do the work {dividing activity by time}, which I didn’t like at first, but gradually, I became able to rest like everyone else {modifying one’s own tendency}. I was frustrated when things didn’t go my way {frustrated when things didn’t go my way}. At such times, I noticed that my emotions changed as I engaged in the work {mood changes while engaging in tasks in front of me}. Working collaboratively with others for the tasks such as taking care of plants, I realized that even if I didn’t like the work at first, I could feel that it was more or less rewarding as I continued to do it. Although being a co-leader was something I hated the most, as I undertook the role, I found that there were things that I could not have done if I was not a co-leader, and I enjoyed it {becoming motivated after getting on with tasks}. I thought I would miss something valuable if I judge without even trying {modifying one’s own tendency}.

#### 3.3.1. Comparison by BDI-II Score Immediately after Bed-Resting

The group of patients whose BDI-II scores improved after bed-resting (16 cases) was compared with the group whose BDI-II scores showed no improvement after bed-resting (3 cases) (Table 7).

The non-improved bed-resting group showed lower percentages in “experience of thinking it through”, “increased desire for activity”, “sense of accomplishment”, and “restoration of healthy sensations”. In the post bed-rest experiences, the non-improved group showed a higher proportion of “identifying maladaptive behavior patterns”, “modifying maladaptive behavior patterns”, and “change in negative emotions”.

#### 3.3.2. Comparison by the Course of BDI-II Scores after Bed-Resting

After bed-resting, two major patterns were found in the progression of BDI-II scores: the group whose BDI-II scores continued to decline (6 cases), and the group whose scores continued to decline while going up and down (13 cases). So, the group whose BDI-II scores continued to decrease after bed-resting was labelled as the decreased group, and the group whose scores lowered while going up and down was labelled as the fluctuating group. These two groups were then compared (Table 7).

The fluctuating group displayed a higher proportion of “experience of getting stuck doing things one’s own way”, “modifying maladaptive behavior patterns”, and a lower proportion of “keeping regular hours”.

## 4. Discussion

### 4.1. Efficacy of Inpatient MT for Depressed Patients

In this study, the BDI-II and HAM-D scores on admission and at discharge were compared in the 19 inpatients with mood disorders who completed MT, and both showed a significant decrease at discharge. However, we could not prove that inpatient MT was effective in improving depression in inpatients with mood disorders because there was no control group, and, the subjects requested inpatient MT on their own, meaning convenience sampling was employed in this study. In addition, various confounding factors, including the Hawthorne effect, were present and could not be adjusted for.

### 4.2. About Isolation Bed-Resting

#### 4.2.1. Quantitative Examination of Depression before and after Bed-Resting

A comparison of BDI-II scores on admission and at the end of bed-resting in the 19 patients who completed the treatment showed a significant decrease in total scores after bed-resting. Of the 19 completers, 3 displayed worsened scores as a result of bed-rest. However, their BDI-II scores at discharge were lower than on admission. Therefore, a worsening BDI-II score after bed-resting could not be considered as an indicator of outcome at discharge.

#### 4.2.2. Qualitative Examination of Depression before and after Bed-Resting

Originally, Morita regarded the significance of bed-resting during inpatient MT as being useful for differential diagnosis, for rest of the body and mind, and for facing anxiety and distress—the experience of immediate release from anguish [1]. In other words, for patients with anxiety disorders, it is considered important to cultivate the attitude of "leaving anxiety as it is without trying to control it." On the other hand, Nakamura [13] stated that for patients with mood disorders, bed-resting was significant for mental and physical rest. The results of this study, however, suggest that there is not only the effect of mental and physical rest, but also an improvement in the depressive state. In addition, looking at the course of the bed-resting experience of patients with mood disorders, many seemed to have gone through the course of “rest of body and mind”, “reflection and regret about the past”, “emergence of boredom”, “increased desire for activity” and “emergence of anxiety about the future”, which is similar to the stepwise phases that anxiety disorder patients follow, in the order of a period of rest, a period of anguish, a period of boredom, and the activation of a desire for life [1].

### 4.3. Qualitative Examination of Factors Contributing to Recovery from Depression in Inpatient MT

#### 4.3.1. Categories of Experiences That Patients Consciously Considered as Opportunities for Improvement and Inflection Points

Among the experiences that contributed to recovery, the experiences that triggered improvement, and the experiences at the inflection point when BDI-II and HAM-D scores improved significantly, were the following six common experiences: “isolation bed-resting of MT”, “getting stuck doing things one’s own way”, “identifying maladaptive behavior patterns”, “modifying maladaptive behavior patterns”, “restoring self-evaluation”, and “change in negative emotions”. These results suggest that these six experiences are important factors in the recovery of patients with mood disorders during inpatient MT.

#### 4.3.2. The Timing of the Experience of Each Category and Relationships among Different Categories of Experiences

In general, patients with typical anxiety disorders follow the process as below during inpatient MT [18]. In the bed-resting period, patients are detached from real life, face anxiety and distress, and become aware of their own desire for activity (desire for life). In the subsequent light work period and work period, they are encouraged to “step into work while being anxious” and are asked to engage in what is in front of them, while feeling anxiety and distress as they do so. Practicing the above process can lead to the demonstration of a desire for life. As they engage themselves in the real work, there is a shift in perception from assuming that their symptoms are special to recognizing that they are a problem that anyone can have, as well as a shift in attitude from trying to eliminate them to acceptance. The treatment then gradually progresses to address the issues of real-life relationships, work methods and so on, and the theme of treatment shifts from “toraware (being fixed on anxiety)” to “living”, which is how patients with anxiety disorders recover.

**Figure 3:**
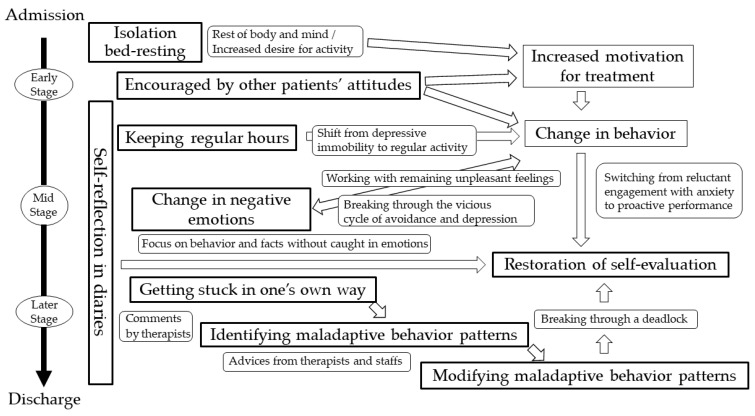
Timing and relationships of experiences contributing to recovery from depression.

Based on the results of this study, subjective experiences that lead to improvement in depressed patients during inpatient MT were categorized using qualitative data analysis methods [17]. The same method was then used to examine the timing and content of the experiences of these categories, and the relationships among the categories were discussed and developed into a storyline, which is shown in Figure 3.

The experience of “isolation bed-resting of MT” helped patients to rest their body and mind, and moreover, increased their desire for activity, which was considered to give impetus to the treatment from the light work period onwards. The experience of “being encouraged by other patients’ attitudes” was often described as an experience in the early stage of inpatient treatment; and the presence of peers who were trying to cure their illnesses together during hospitalization encouraged the patients, enhanced their motivation for treatment, and facilitated their behavioral change. In addition, “keeping regular hours” was also experienced in the earlier part of hospitalization, and supported behavioral change. While many patients were prone to think “I don’t feel well, so I will take a break”, they started to think, “I don’t feel well, but it’s time for work, so I have no choice but go out to work”. Thus, the patients showed a behavioral change with inpatient MT, which is similar to the behavioral activation (BA) [19] proposed by Lewinsohn, P.M. et al. in the 1970s, in that it promotes behavioral change in patients. While the timing of the start of behavioral activation is not clearly defined in BA, inpatient MT is characterized by the gradual activation of behaviors, from the period during which patients are required to stay in bed to rest their body and mind and develop an increased desire for activity, to the periods of light work and work.

A “change in negative emotions” was often experienced in the middle stage of hospitalization. It was often obtained by means of the work they engaged in while dealing with unpleasant emotions. This can be an experience involving restoring the fluidity of the mind, as the mind that has been stuck in a vicious cycle of depression [20] moves along with the actions associated with work. Patients had the experience of “restoring self-evaluation” from the middle to the end of hospitalization via a process in which they engaged anxiously or begrudgingly in the work in the beginning and gradually became more proactive, gaining a sense of accomplishment which boosted their self-confidence. “Self-reflection in a diary” was experienced throughout the hospitalization period, helping to restore self-evaluation by focusing not only on negative feelings but also on the patient’s own actions and facts of life, and also providing opportunities for being encouraged by the therapist’s comments and reflecting on their own tendencies. In the mid to late stage of hospitalization, when self-evaluation had recovered to some extent, the patients experienced “getting stuck doing things one’s way” in work and interpersonal situations; and by taking advice from therapists and staff, they gained the experience of “identifying maladaptive behavior patterns” and “modifying maladaptive behavior patterns”.

#### 4.3.3. Comparison of Categories by BDI-II Score Immediately after Bed-Resting

In the improved bed-resting group, the patients rested their physical and mental state, regained a healthy sense of their bodies, directed their interest toward the outside world, acknowledged fresh perceptions, and had a heightened desire for activity. Many of them also had experiences of “thinking it through” and they reported that they had reached a state of mind departing from the ruminations associated with depressive thoughts. Thus, for the improved bed-resting group, it is considered that bed-resting is not just a simple break, but rather it is useful in improving the physical dimension of recovery as well. On the other hand, the non-improved bed-resting group had lower percentages of “thinking it through”, “increased desire for activity” and “restoration of healthy sensation”. As for the experiences after bed-resting, all patients in the non-improved bed-resting group reported the experience of a “change in negative emotions”, “identifying maladaptive behavior patterns” and “modifying maladaptive behavior patterns”. Based on these findings, depressed patients who cannot change their negative emotions through rest alone might need to experience changes in negative emotions through work (or activity) to recover from depression.

#### 4.3.4. Comparison of Categories by the Course of BDI-II Scores after Bed-Resting

The fluctuating group had higher percentages of “experiences of getting stuck doing things one’s way” and “experiences of modifying maladaptive behavior patterns”, and lower percentages of “keeping regular hours”. This might mean that, for depressed patients whose depressive symptoms do not improve simply by adjusting their daily lives, “experience of getting stuck doing one’s way” is important for recovery. In the fluctuating group, BDI-II scores worsened each time they experienced “getting stuck doing things one’s way”, but each time this happened, it was considered to have led to the experience of “modifying maladaptive behavior patterns”. Thus, it might be important to experience “getting stuck doing things one’s way” in order to modify one’s maladaptive behavior patterns.

### 4.4. Specific Recovery Mechanisms for Depression in Inpatient MT

Based on clinical observations of 899 patients with manic–depressive psychoses, Kraepelin stated that “usually a single illness would last six to eight months [21]. Normally, all symptoms disappear completely”. Based on the above view that depression is essentially an illness that leads to spontaneous recovery, the recovery mechanisms of depression will be discussed in this section by distinguishing the nonspecific (common) recovery mechanism from specific recovery mechanisms.

The nonspecific recovery mechanism is a common mechanism regardless of the case, and is activated not only by psychological triggers but also by physiological and physical triggers. Janet suggested that the treatment of depression should begin with the application of the method of psychological power-saving and rest, and that, from this rest, an upsurge can occur [22]. Thus, the recovery mechanism of depression was understood as progressing from rest of the body and mind to an upsurge. The therapy referred to here can be said to have a stimulating effect on behavior, including uplifting morale, which may be considered equivalent to the occupational therapy, behavioral activation and exercise therapy of modern times. According to Stassen et al., antidepressants also act as recovery triggers, without altering the natural recovery process pattern of depression [23]. Specific recovery mechanisms, on the other hand, are recovery processes that progress by modifying or eliminating impediments to recovery that are specific to patient types or to each patient.

Inpatient MT is based on nonspecific recovery mechanisms, and in the process of recovery, specific recovery mechanisms are thought to be synergized in terms of modifying psychological factors (“the way things should be”) that impede recovery [13].

Among the categories of experiences that contributed to recovery from depression identified in this study, rest of the body and mind in “bed-resting experience”, “keeping regular hours”, “self-reflection in a diary”, and “being encouraged by other patients’ attitudes” were considered nonspecific recovery mechanisms, while experiences of “bed-resting” other than rest of the body and mind, “getting stuck doing things one’s own way”, “identifying maladaptive behavior patterns”, “modifying maladaptive behavior patterns”, “restoring self-evaluation”, and “change in negative emotions” were considered to be specific recovery mechanisms with inpatient MT (Table 8).

### 4.5. The Role of Nurses in the Experience of Inpatient MT Contributing to the Recovery of Depression

During inpatient MT, patients undergo a variety of experiences, and nurses play an important role in sharing life and work situations with patients. In general, the role of the nurse in inpatient MT is considered to mainly involve “watching over” and “intervention”. In other words, it is important for nurses to watch over and encourage patients in a receptive manner, rather than directly reaching out to them, and to be involved in their lives from the same perspective, understanding their struggles and strengths, which can easily lead to progress in treatment. In addition, according to Nakaema et al. [24], the “nursing presence there” included in watchful waiting leads to an attitude of sharing experiences with the patient and exploring them together. It is assumed that many of the experiences that contributed to recovery from depression identified in this study also involved the role of nurses. For example, the sense of safety that comes from being watched over by the nurse in daily life and in the workplace, and the fact that the nurse has shared experiences with the patient, may have been related to “modifying maladaptive behavior patterns” and other experiences. Although this study was not able to clearly investigate the role of nurses in experiences that contributed to recovery from depression, we would like to consider this in the future.

### 4.6. Limitations of This Study

Five limitations of this study were identified.

1. A total of 414 patients received inpatient MT during the period of data collection, of whom 90 had a diagnosis of a mood disorder. However, the number of subjects was reduced to 24 as they were selected strictly to only include patients aged 20 to 65 years and excluding those with other psychiatric and behavioral disorders, such as anxiety disorders, personality disorders, and developmental disorders, as well as those who were readmitted to the hospital. The sample size of this study was small, and it needs to be expanded upon in the future.
2. In general, patients with mood disorders who are candidates for inpatient MT have already received general treatment, including rest and medication, which may have alleviated their acute depression to some extent, but many of them are in a prolonged state of mild depression [20]. In fact, this study included patients with mood disorders who had undergone inpatient MT, and most of them had persistent disorders (mean duration of illness: 8.27 years). Therefore, this study could not investigate the treatment process of the acute phase of patients with mood disorders.
3. The subjects of this study were patients who visited a university hospital in Tokyo, Japan, and voluntarily requested MT. The investigation was conducted at only one institution and may have different characteristics from studies conducted at non-university hospitals.
4. Only those who completed the treatment could be interviewed at the time of discharge, and not those who discontinued treatment.
5. Regarding pharmacotherapy, no significant difference in the use of antidepressants was found before and after hospitalization. However, other medications such as mood stabilizers and anxiolytics were also used at times, the effects of which could not be investigated.

## 5. Conclusions

In this study, nine categories of experiences that contributed to recovery from depression were identified among those who completed inpatient MT: “isolation bed-resting of MT”, “getting stuck doing things one’s way”, “identifying maladaptive behavior patterns”, “modifying maladaptive behavior patterns”, “restoring self-evaluation”, “change in negative emotions”, “keeping regular hours”, “self-reflection in a diary”, and “being encouraged by other patients’ attitudes”. In particular, of these nine categories, the first six experiences were considered as the key experiences for recovery.

Furthermore, the recovery process of patients with mood disorders during inpatient MT showed that there were two patterns: one group showed steady improvement, and the other group showed improvement after repeated deterioration and improvement. In addition, experiences of “bed-resting” other than rest of the body and mind, “getting stuck doing things one’s way”, “identifying maladaptive behavior patterns”, “modifying maladaptive behavior patterns”, “restoring self-evaluation”, and “change in negative emotions” were considered as the recovery mechanisms specific to inpatient MT.

## Figures and Tables

**Table 1:** Backgrounds of subjects.

Item	Value	
	19 Completers	5 Discontinuers
Average Age ± SD (years)	41.3 ± 9.51	45.6 ± 9.93
Sex (F/M)	10/9	1/4
Diagnosis		
Depressive episode	1 case	1 case
Recurrent depressive disorder	10 cases	3 cases
Bipolar–affective disorder (all type II)	8 cases	1 case
Average duration of illness, Average ± SD (years)	8.27 ± 6.83	3.35 ± 5.69
Average HAM-D score on admission, Average ± SD	13.37 ± 5.86	12.40 ± 3.14
Average BDI-II score on admission, Average ± SD	25.53 ± 9.18	26.80 ± 5.04
Average antidepressant on admission: imipramine equivalent, Average ± SD (mg)	179.2 ± 134.8	121.0 ± 53.1
Average antidepressant at discharge: imipramine equivalent, Average ± SD (mg)	163.4 ± 126.4	176.0 ± 76.5
Average duration of isolation bed-resting period, Average ± SD (days)	7.16 ± 0.67	
Average duration of light work period, Average ± SD (days)	5.11 ± 0.45	
Average duration of intensive work period, Average ± SD (days)	68.89 ± 32.85	
Average duration of preparation for daily living period, Average ± SD (days)	13.42 ± 10.19	
Average Length of Hospitalization, Average ± SD (days)	94.58 ± 39.95	

SD: standard deviation. BDI-II: Beck Depression Inventory Second Edition. HAM-D: Hamilton Depression Rating Scale.

**Table 2:** Average and standard deviation of HAM-D subscores before and after inpatient treatment.

	Admission Average ± SD	Discharge Average ± SD
1. Depressed mood	1.42 ± 1.14	0.21 ± 0.41 **
2. Work and Activities	1.84 ± 0.99	0.47 ± 0.94 **
3. Genital Symptoms	0.53 ± 0.68	0.16 ± 0.36 **
4. Somatic Symptoms–Gastrointestinal	0.37 ± 0.58	0.21 ± 0.52
5. Loss of Weight	0.21 ± 0.41	0.05 ± 0.22
6. Insomnia Early	0.74 ± 0.85	0.05 ± 0.22 **
7. Insomnia Middle	0.79 ± 0.77	0.11 ± 0.31 **
8. Insomnia Late	0.37 ± 0.58	0.26 ± 0.44
9. Somatic symptoms–General	0.95 ± 0.39	0.16 ± 0.36 **
10. Feelings of Guilt	1.05 ± 0.89	0.05 ± 0.22 **
11. Suicide	0.68 ± 0.80	0.05 ± 0.22 **
12. Anxiety Psychic	1.21 ± 1.10	0.32 ± 0.46 **
13. Anxiety Somatic	0.84 ± 0.81	0.16 ± 0.36 **
14. Hypochondriasis	0.37 ± 0.58	0.16 ± 0.49
15. Insight	0.00 ± 0.00	0.00 ± 0.00
16. Retardation	0.58 ± 0.67	0.05 ± 0.22 **
17. Agitation	0.21 ± 0.52	0.00 ± 0.00
18. Diurnal Variation	0.63 ± 0.48	0.16 ± 0.36 **
19. Depersonalization and Derealization	0.32 ± 0.57	0.00 ± 0.00 *
20. Paranoid Symptoms	0.05 ± 0.22	0.00 ± 0.00
21. Obsessional and Compulsive Symptoms	0.21 ± 0.52	0.00 ± 0.00
Total 21-Item Hamilton Depression Score	13.37 ± 5.86	2.63 ± 2.03 **

SD: Standard deviation, ** *p* < 0.01, * *p* < 0.05, paired-samples *t*-test.

**Table 3:** Average and standard deviation of BDI-II subscores at admission, at the end of bed-resting, and at the time of discharge.

	Admission Average ± SD	End of Bed-Rest Average ± SD	Discharge Average ± SD
1. Sadness	1.05 ± 0.69	0.74 ± 0.55	0.26 ± 0.44 *
2. Pessimism	1.58 ± 0.59	0.74 ± 0.71 *	0.26 ± 0.44 *
3. Past Failure	1.42 ± 0.88	1.05 ± 0.76 *	0.47 ± 0.60 *
4. Loss of Pleasure	1.68 ± 0.80	1.05 ± 0.83 *	0.26 ± 0.44 *
5. Guilty Feelings	0.79 ± 0.77	0.47 ± 0.50	0.05 ± 0.22 *
6. Punishment Feelings	0.32 ± 0.46	0.37 ± 0.81	0.11 ± 0.31
7. Self-Dislike	1.53 ± 0.82	1.32 ± 0.80	0.32 ± 0.65 *
8. Self-Criticism	1.42 ± 0.88	1.00 ± 0.79 *	0.32 ± 0.57 *
9. Suicidal Thoughts or Wishes	0.84 ± 0.49	0.63 ± 0.48	0.16 ± 0.36 *
10. Crying	1.00 ± 1.03	0.79 ± 0.69	0.16 ± 0.36 *
11. Agitation	1.05 ± 0.60	0.74 ± 0.44	0.21 ± 0.41 *
12. Loss of Interest	1.32 ± 0.80	0.42 ± 0.49 *	0.21 ± 0.41 *
13. Indecisiveness	1.63 ± 0.93	1.11 ± 0.72 *	0.21 ± 0.41 *
14. Worthlessness	1.32 ± 0.73	1.11 ± 0.85	0.26 ± 0.64 *
15. Loss of Energy	1.79 ± 0.61	1.05 ± 0.76 *	0.32 ± 0.46 *
16. Changes in Sleeping Pattern	1.11 ± 0.79	1.26 ± 1.02	0.53 ± 0.68
17. Irritability	0.68 ± 0.98	0.58 ± 0.67	0.16 ± 0.49 *
18. Changes in Appetite	0.68 ± 0.73	0.74 ± 0.78	0.42 ± 0.82
19. Difficulty Concentrating Difficulty	1.74 ± 0.78	1.00 ± 0.73 *	0.21 ± 0.52 *
20. Tiredness or Fatigue	1.63 ± 0.87	1.16 ± 0.93	0.53 ± 0.50 *
21. Loss of Interest in Sex	0.95 ± 1.05	0.68 ± 0.98	0.21 ± 0.52 *
Total Score	25.53 ± 9.18	18.00 ± 9.53 *	5.63 ± 6.63 *

SD: Standard deviation, * *p* < 0.05, Repeated analysis of variance (ANOVA). *p*, Bonferroni-adjusted *p*-value.

**Table 4:** Summary of experiences that contributed to recovery from depression.

Category of Experience	Code	Example of Sentence Segments (Subjective Experiences Leading Improvement)	Case
Isolation bed-resting during Morita Therapy	Bed-resting	“The experience of bed-resting helped me in my recovery.” (all cases), {bed-resting}	19
Getting stuck doing things one’s own way	Troubled by interpersonal relationships; irritated by disagreement; irritated by wanting something from others; taking a break due to being depressed; irritated with not getting one’s way; taking a break due to being tired; overworking and getting sick; wanting to run away; suffering from doing as one wishes; thinking one is doomed; tired by trying to do it alone; sad at not getting one’s way	“I was troubled by the relationship with people I didn’t like” (Case 1), {troubled by interpersonal relationships}. “I was feeling very depressed and was going to take a break.” (Case 7), {trying to take a break from work due to being depressed}. “I had to work harder than others as I felt anxious if I didn’t do so, but I tried too hard and got sick.” (Case 13), {overworking and getting sick}.	11
Identifying maladaptive behavior patterns	Realizing one’s issue; realizing one’s tendency; reflecting on one’s behavior; reflecting on one’s style; facing oneself	“I was involved with other patients and nurses every time I worked, and at first, I strongly felt that I was interfered by them all of the time, but it gave me an opportunity to reflect on my style such as wanting to do things my way, and not consulting others or not being able to do so” (Case 9), {reflecting on one’s style}, {realizing one’s own tendency}. “I realized that I had been pushing myself to do what I thought I had to do this way. I realized that I had been doing things according to my own values without considering others” (Case 15), {realizing one’s own tendency}.	14
Modifying maladaptive behavior patterns	Modifying one’s tendency; tackling one’s problem	“With the advice of a staff, I was able to take care of what was in front of me one at a time, consulting with other patients, whereas I would have usually done the work all by myself” (Case 3), {modifying one’s own tendency}. “I became aware of my own tendencies such as not being able to give help signs and not being able to ask for help, and I gradually became able to decide who, what and how to ask for help to some extent, or how much I should meddle” (Case 11), {modifying one’s own tendency}.	11
Restoring self-evaluation	Gaining confidence by accomplishing a task; gaining confidence by becoming able to do more things; gaining confidence that one could do it; gaining confidence by being acknowledged by everyone; gaining confidence by working proactively; feeling that one is not hopeless; becoming able to see what could be done; anxiety alleviates after getting on with work	“I felt that I was able to prepare for the event and to lead the plants group which gave me confidence. I could feel that I was not that bad” (Case 16), {gaining confidence by doing something}, {feeling I am not a failure}. “I used to think I was a person who could not do anything, but now I feel that I can do things little by little. I feel that I can do things that I am not good at if I try. I came to think that I should look at not only what I can’t do, but also what I can do. I was able to gain confidence for the first time after having experienced so many things here” (Case 18), {beginning to see what one can do}, {gaining confidence as one can do more things}.	13
Change in negative emotions	Bad feelings change as you work; mood changes while engaging in tasks in front of me; mood flows by moving by time; forgetting other things while working silently; becoming motivated after getting on with tasks	“There was a time when I was working, and before I knew it, bad feelings became lighter. After this experience, I was no longer completely immobilized by bad feelings.” (Case 1), {bad feelings change while working}. “I was not motivated about work until I was hospitalized. The same was true for the work in the ward, but as I got grips with it, I became enthusiastic and was motivated naturally.” (Case 2), {mood changes while getting on with tasks}. “During my hospitalization, I felt a strong sense of anticipatory anxiety and irritation at the thought of what was to come, but somehow it did not bother me as I was working on tasks.” (Case 5), {bad feelings change while working}.	12
Keeping regular hours	Delimiting by time; set task and time; keeping life’s rhythm; rest at a break; not staying in bed even if feeling unwell	“It was easy for me to have a rhythm as there was a set task and a set time.” (Case 4), {set task and time}. “I like the environment where I could not do more even if I wanted to, since every work had certain time frame. In a way, I could give up and take a break.” (Case 13), {delimiting by time}.	8
Self-reflection in a diary	Reflecting on behaviors in a diary; reflecting on oneself in a diary; reflecting on feelings in a diary; being alert enough to write a diary	“Looking back on my behaviors in my diary, I could see that I had done something.” (Case 1), {reflecting on one’s behaviors in a diary}. “By keeping a diary, I could look back and realize that I had been troubled by the same things before.” (Case 15), {reflecting on oneself in a diary}.	5
Being encouraged by other patients’ attitudes	Being inspired by other patients; being encouraged by other patients; being cheered up by other patients	“I was encouraged by the hard work of other patients.” (Case 5), {encouraged by other patients}. “I saw a very talented patient make mistakes, and it made me think that it was okay to make mistakes.” (Case 16), {empowered by other patients}.	4

**Table 5:** Summary of isolation bed-resting experiences.

Category	Timing of Experience	Code	Example of Sentence Segments (Subjective Experiences Leading Improvement)	Case
Rest of body and mind	Early Bed-Resting~Whole Periods	Rest; rest away from usual place; rest leaving usual roles; better sleep; lighter body; blood circulated to brain; recovery of taste; recovery from heavy-headedness; feeling better; depression gone; something gone	“I was always in a rush to do something even when I stayed at home on leave as I felt I was bothering my family, but I could rest fully as I was in an environment where I could not do anything while bed-resting” (Case 13), {rest away from usual place}, {rest leaving usual roles}. “I began to sleep well” (Case 12), {improved sleep}. “My head is no longer heavy and I feel refreshed” (Case 8), {alleviation of heavy-headedness }.	19
Reflection and regret about the past	Early Bed-Resting	Regret about the past; think about the past; reflection on the past; conflict with oneself; self-criticism; conflict about treatment; regret about past treatment; sorry for one’s family; worried about one’s family; sorry for the people at work; crying	“In the first half of the bed-resting period, I agonized over the things I had done in the past, repeatedly having reflections and pessimistic thoughts.” (Case 9), {thinking about the past}, {reflection on the past}. “From the third or fourth day of bed-resting, I had a difficult time, regretting about the past.” (Case 2), {regret about the past}.	12
Anxiety about the future	Late Bed-Resting~Whole Periods	Anxiety about work ahead; anxiety about future relationships; anxiety about future treatment; anxiety about returning to society; anxiety about life after discharge; vague anxiety about the future; anxiety about one’s body	“In the latter half of the bed-resting period, I was anxious about whether I could do the work after rising from the bed-resting and whether I could reintegrate into society.” (Case 2), {anxiety about future work}, {anxiety about reintegration into society}. “I had a hard time always worrying about the future such as my life after discharge.” (Case 17), {anxiety about life after discharge}.	13
Thinking thoroughly	Mid Bed-Resting	Thinking through the past; thinking through; thinking it through to find that there was no answer; thinking through one’s treatment concerns; what will be will be; facing oneself; making up one’s mind to do what could be done; crying as much as one can; accepting treatment	“I felt sorry for causing trouble to everyone, but I thought that getting better and doing things at my own pace was the only way to go.” (Case 13), {making up one’s mind to do what could be done}. “I was anxious about whether I would be able to fit in with everyone after the bed-resting, but I thought it was no use thinking about it anymore.” (Case 9), {thinking it through to find that there was no answer}.	11
Emergence of boredom	Mid Bed-Resting~Late Bed-Resting	Becoming bored; sense of boredom intensified; having nothing to do	“I became bored from about the 5th day, and the 6th day was too boring and stressful” (Case 14), {becoming bored}, {sense of boredom intensified}.	10
Increased desire for activity	Late Bed-Resting	Wanting to look outside; wanting to go outside; looking forward to going outside; wanting to move one’s body; wanting to join the work; wanting to join the group; wanting to do various things; wanting to take a bath; longing for physical activity; being frustrated at not being able to move; moving one’s body knowing it is forbidden; talking to others knowing it is forbidden	“In the latter half of the bed-resting period, I was eager to go outside and look at the view.” (Case 1), {wanting to go outside}, {wanting to look outside}. “In the latter half of the bed-resting, I wanted to try various activities.” (Case 12), {wanting to do various things}. “From about the 5th day of bed-resting, I wanted to move my body and go outside for fresh air.” (Case 20), {wanting to move one’s body}, {wanting to go outside}.	12
Sense of accomplishment	Immediately after Bed-Resting	Sense of accomplishment by completing a task; sense of accomplishment	“I felt a sense of accomplishment after completing the bed-resting” (Case 5), {sense of accomplishment}. “I also felt a sense of accomplishment when I finished.” (Case 7), {sense of accomplishment}.	3
Restoration of healthy sensations	1–2 days after Bed-Resting	Feeling good outside; sunshine felt good; weather is nice and pleasant; bathing felt good; feels good to move; fresh perception; fresh feeling; interested in the work; pleased to join the work	“It felt so good to take a bath, and I was surprised that soap was so fragrant” (Case 10), {bathing felt good}. “I felt good to be outside in the fresh air” (Case 1), {feeling good outside}. “Everything outside appeared fresh” (Case 15), {fresh perception}. “I was happy to look at beautiful leaves and smell herbs” (Case 5), {fresh perception}. “It was fresh and pleasant to walk after a long time” (Case 3), {fresh perception}. “It was fun to move my body” (Case 22), {physical activity feels good}.	17
Recovery of concentration	3–5 days after Bed-Resting	Concentrating on wood carving; immersed in wood carving; wood carving is fun; realizing that one can move; concentrating on light work; time passes by quickly	“I was able to concentrate and enjoy wood carving more than I expected” (Case 9), {concentrating on wood carving}.	19

**Table 6:** Experiences as a trigger for improvement and experiences at the inflection point of improving BDI-II and HAM-D scores.

Category of Experience	Experience as Trigger for Improvement	Experience at Inflection Point of Improving BDI-II and HAM-D Scores
Isolation bed-resting of Morita Therapy	15 cases	14 cases
Getting stuck doing things one’s own way	9 cases	8 cases
Identifying maladaptive behavior patterns	7 cases	8 cases
Modifying maladaptive behavior patterns	5 cases	7 cases
Restoring self-evaluation	7 cases	9 cases
Change in negative emotions	8 cases	5 cases
Keeping regular hours	No cases	No cases
Self-reflection in a diary	No cases	No cases
Being encouraged by other patients’ attitudes	No cases	No cases

**Table 7:** Comparison of experiences by BDI-II score immediately after bed-resting and the progression of BDI-II scores after bed-resting.

Subjective Experiences Contributing to Improvement		BDI-II Score at End of Bed-Rest				Progression of BDI-II			
		Improved		Non-Improved		Decreased		Fluctuated	
		16 Cases	%	3 Cases	%	6 Cases	%	13 Cases	%
Experiences during Bed-Resting	Rest of body and mind	16	100.0	3	100.0	6	100.0	13	100.0
	Reflection and regret about the past	9	56.3	3	100.0	5	83.3	7	53.8
	Anxiety about the Future	10	62.5	3	100.0	4	66.7	9	69.2
	Thinking thoroughly	11	68.8	0	0.0	3	50.0	8	61.5
	Emergence of boredom	8	50.0	2	66.7	3	50.0	7	53.8
	Increased desire for activity	12	75.0	0	0.0	4	66.7	8	61.5
	Sense of accomplishment	3	18.8	0	0.0	2	33.3	1	7.7
	Restoration of healthy sensations	16	100.0	1	33.3	5	83.3	12	92.3
	Recovery of concentration	16	100.0	3	100.0	6	100.0	13	100.0
Experiences after Bed-Resting	Getting stuck doing things one’s own way	9	56.3	2	66.7	1	16.7	10	76.9
	Identifying maladaptive behavior patterns	11	68.8	3	100.0	4	66.7	10	76.9
	Modifying maladaptive behavior patterns	8	50.0	3	100.0	2	33.3	9	69.2
	Restoring self-evaluation	11	68.8	2	66.7	4	66.7	9	69.2
	Change in negative emotions	9	56.3	3	100.0	4	66.7	8	61.5
	Keeping regular hours	7	43.8	1	33.3	4	66.7	4	30.8
	Self-reflection in a diary	4	25.0	1	33.3	0	0.0	5	38.5
	Encouraged by other patients’ attitudes	4	25.0	0	0.0	1	16.7	3	23.1

**Table 8:** Specific recovery mechanisms for patients with mood disorders during inpatient Morita therapy.

Specific Recovery Mechanisms in Inpatient Morita Therapy	Nonspecific Recovery Mechanisms in Inpatient Morita Therapy
Experiences of bed-resting other than rest of the body and mind	Rest of the body and mind in bed-resting experience
Getting stuck doing things one’s own way	Keeping regular hours
Identifying maladaptive behavior patterns	Self-reflection in a diary
Modifying maladaptive behavior patterns	Being encouraged by other patients’ attitudes
Restoring self-evaluation	
Change in negative emotions	
Change in negative emotions	

## Data Availability

The data that support the findings of this study are available upon request from the corresponding author. The data are not publicly available due to privacy or ethical restrictions.
